# Editorial: Sex differences in cerebrovascular diseases

**DOI:** 10.3389/fneur.2022.1128177

**Published:** 2023-01-10

**Authors:** Christine Kremer, Svetlana Lorenzano, Christina Kruuse

**Affiliations:** ^1^Neurology Department, Skåne University Hospital Malmö, Malmö, Sweden; ^2^Department of Clinical Sciences, Lund University, Lund, Sweden; ^3^Department of Human Neurosciences, Sapienza University of Rome, Rome, Italy; ^4^Stroke Unit and Neurovascular Research Unit, Neurology Department, University of Copenhagen Herlev Gentofte Hospital, Hellerup, Denmark

**Keywords:** stroke, prevention, treatment, outcome, sex differences

Sex difference in the occurrence, detection, and treatment of cerebrovascular disease is being increasingly recognized. Indeed, recent studies have focused on how the etiology, course, and outcome of cerebrovascular disease may differ in men and women (1). Moreover, the effectiveness and safety of primary prevention, acute treatments, and secondary prevention of stroke may be affected by factors related to sex (2). Continued attention and focused research on these differences will allow us to have a better insight into the underlying pathophysiological, socioeconomic, and organizational aspects and improve stroke outcomes for men and women. Unfortunately, a significantly lower number of women over the years have been included in clinical stroke trials, leading to a limited amount of evidence for many stroke treatments in women.

In the Research Topic, “*Sex Differences in Cerebrovascular Diseases*,” clinical research and reviews on different aspects of epidemiology, prevention, treatment, and outcome in men and women with cerebrovascular diseases during their lifespan are presented. Regarding the reproductive lifespan of women, it has been shown that early menarche is associated with a higher percentage of cerebrovascular events in later life (3). Additionally, during pregnancy and postpartum, the risk of stroke is increased. Contrary to the general decreasing trend of stroke incidence in the general population of western countries, stroke in pregnancy is increasing, as shown in a review of seven studies by Ijäs et al.. This is likely because of generally advancing maternal age, more prevalent comorbidities, and the presence of vascular risk factors, such as hypertension (Ijäs et al.). This stresses the importance of prospective studies and international registries, such as the Stroke in Pregnancy and Postpartum Study (SiPP) (4).

In another article in the collection by Norman et al. general sex differences in a younger age group and in trends in stroke incidence were analyzed using a large registry. Younger men appeared to have more conventional vascular risk factors and higher case fatality than younger women (Norman et al.).

Notwithstanding, others found that younger women taking oral contraceptives had a higher stroke risk, which was accentuated in women with a high BMI and increased 10-fold in women with migraines who also smoked (5).

Physical inactivity resulting in a higher BMI, a higher risk for metabolic syndrome, and higher blood pressure have become major challenges for stroke prevention, not only in the general population in western countries but also in relation to sex. Indeed, while on one side it could be concluded that physical inactivity has an impact on stroke severity in both sexes, stroke severity seems to be worse in women (Salmantabar et al.).

There are conflicting results regarding primary prevention with acetyl acetylic acid (ASA) in men and women. In the review by Gdovinova et al., ASA was effective in women with no increased risk of hemorrhagic stroke; however, they found an increase in general systemic bleeding risk. These data are indicative of a potential difference in antiplatelet treatment effect between sexes, which should be further investigated (Gdovinova et al.).

Stroke etiology was reported to differ between men and women, with more men showing large artery atherosclerosis and women having more cardiac embolic strokes due to an increased frequency of atrial fibrillation. In an analysis of systematic reviews, it was observed that there was a male preponderance in displaying cerebral small vessel disease-related ischemic stroke and that men tended to present more moderate-to-severe cerebral small vessel disease (Jiménez-Sánchez et al.).

Conflicting results were found in the study by Pavlovic et al., in which women with previous lacunar stroke had a more severe cerebral small vessel disease than men, particularly white matter hyperintensity, and seemed to develop cognitive impairment more frequently than their male counterparts. However, this association with sex was dependent on the occurrence of depression and the severity of white matter hyperintensities and could not be explained by differences in common vascular risk factors, which were not significantly different between women and men. This has implications for further clinical and translational research and organizational aspects of patient care (Pavlovic et al.).

Vascular risk factors seem to have a relevant impact, particularly in women during and after menopause. Women showed an increased stroke risk partly due to a change in arterial stiffness and a higher risk of hypertension (6). There are conflicting results regarding stroke risk and hormonal therapy. In the recently published guidelines of the European Stroke Organization, it was concluded that low to very low evidence on the subject was found, hence only a weak recommendation against the use of hormonal therapy during menopause could be given (7).

Additionally, in the acute treatment of stroke, sex may have an important role. Two articles focused on pre-hospital identification of stroke and possible delays in women. As shown by Walter et al., awareness of stroke symptoms and pre-notification patterns differ between men and women (Walter et al.). Women were well-informed about stroke symptoms but they showed less self-awareness when suffering a stroke. Women were more likely to live alone when older, which could lead *per se* to delays in reporting and notification. Additionally, once admitted, do not resuscitate orders were given more frequently for women than for men, a finding that needs to be explored further.

As reported with myocardial infarction, women who had a stroke reported more uncommon symptoms that could be misdiagnosed as stroke mimics (Eddelien et al.). The less identifiable stroke symptoms can make the timely and correct diagnosis of a stroke more difficult and challenging, which could delay acute treatment with intravenous thrombolysis and/or mechanical thrombectomy within the recommended therapeutic time windows.

Stroke outcomes following acute endovascular treatment with mechanical thrombectomy may be sex related. In stroke patients treated with mechanical thrombectomy, women show better collateral flow but at the same time worse outcomes (Lagebrant et al.). These discrepancies should be further investigated.

In the observational study on intracerebral hemorrhage, an analysis of outcomes after intracerebral bleeding while taking oral anticoagulants in 226 men and 176 women showed that women had lower odds of receiving reversal agents and a decision of do not resuscitate, but when surviving had a similar outcome as men at 3 months. Interestingly, stroke-associated risk factors, such as smoking and alcohol intake, were less frequent in women than men but women were older and more dependent according to the modified Rankin Scale (Grundtvig et al.).

A retrospective study on 287 women and men with subarachnoid hemorrhage by Cai et al. reported that women had worse outcomes after subarachnoid hemorrhage and more post-interventional ischemic complications than men (Cai et al.). The reasons for this difference in outcome between sexes are yet to be understood. A worse short-term outcome after stroke in women, in general, was also found by Eren et al. in a retrospective cross-sectional study of 611 female and 683 male stroke patients (Eren et al.). In summary, articles included in this Research Topic showed that numerous sex differences in cerebrovascular diseases exist and impact the diagnosis and treatment in both men and women.

Current evidence suggests that a more tailored and individualized approach to these diseases in both sexes is needed. Sex-specific analyses must be performed. In future randomized controlled trials, more women of all age groups must be included to provide more reliable evidence for stroke treatment in both men and women.

## Author contributions

CKre wrote the draft. All authors contributed to review. All authors contributed to the article and approved the submitted version.
